# Early life nutritional imbalance impairs colonic epithelial regeneration through gut microbiota dysbiosis and metabolic suppression

**DOI:** 10.1093/ismejo/wrag135

**Published:** 2026-05-29

**Authors:** Yimin Zhuang, Zhiyu Huang, Shuai Liu, Yiming Xu, Guobin Hou, Duo Gao, Tianyu Chen, Boyan Ma, Wen Jiang, Jingtao You, Mengmeng Li, Wei Wang, Shengli Li, Zhijun Cao

**Affiliations:** State Key Laboratory of Animal Nutrition and Feeding, International Calf and Heifer Organization, College of Animal Science and Technology, China Agricultural University, Beijing 100193, China; College of Animal Science and Technology, Yangzhou University, Yangzhou, Jiangsu 225009, China; State Key Laboratory of Animal Nutrition and Feeding, International Calf and Heifer Organization, College of Animal Science and Technology, China Agricultural University, Beijing 100193, China; State Key Laboratory of Animal Nutrition and Feeding, International Calf and Heifer Organization, College of Animal Science and Technology, China Agricultural University, Beijing 100193, China; State Key Laboratory of Animal Nutrition and Feeding, International Calf and Heifer Organization, College of Animal Science and Technology, China Agricultural University, Beijing 100193, China; Inner Mongolia Youran Dairy Co., Ltd., Hohhot, Inner Mongolia 010080, China; State Key Laboratory of Animal Nutrition and Feeding, International Calf and Heifer Organization, College of Animal Science and Technology, China Agricultural University, Beijing 100193, China; State Key Laboratory of Animal Nutrition and Feeding, International Calf and Heifer Organization, College of Animal Science and Technology, China Agricultural University, Beijing 100193, China; College of Animal Science and Technology, Beijing University of Agriculture, Beijing 102206, China; State Key Laboratory of Animal Nutrition and Feeding, International Calf and Heifer Organization, College of Animal Science and Technology, China Agricultural University, Beijing 100193, China; State Key Laboratory of Animal Nutrition and Feeding, International Calf and Heifer Organization, College of Animal Science and Technology, China Agricultural University, Beijing 100193, China; State Key Laboratory of Animal Nutrition and Feeding, International Calf and Heifer Organization, College of Animal Science and Technology, China Agricultural University, Beijing 100193, China; College of Animal Science, Xinjiang Agricultural University, Urumqi, Xinjiang Uygur Autonomous Region 830052, China; State Key Laboratory of Animal Nutrition and Feeding, International Calf and Heifer Organization, College of Animal Science and Technology, China Agricultural University, Beijing 100193, China; State Key Laboratory of Animal Nutrition and Feeding, International Calf and Heifer Organization, College of Animal Science and Technology, China Agricultural University, Beijing 100193, China; State Key Laboratory of Animal Nutrition and Feeding, International Calf and Heifer Organization, College of Animal Science and Technology, China Agricultural University, Beijing 100193, China; State Key Laboratory of Animal Nutrition and Feeding, International Calf and Heifer Organization, College of Animal Science and Technology, China Agricultural University, Beijing 100193, China; State Key Laboratory of Animal Nutrition and Feeding, International Calf and Heifer Organization, College of Animal Science and Technology, China Agricultural University, Beijing 100193, China; College of Animal Science and Technology, Beijing University of Agriculture, Beijing 102206, China

**Keywords:** microbial ecosystem, malnutrition, dietary imbalance, short chain fatty acids, gut development

## Abstract

Childhood malnutrition represents a major global health burden that extends beyond caloric deficiency to include dietary imbalance. The early-life gut microbiome functions as a dynamic ecosystem whose structure and metabolic output are intimately linked to host development; yet, how nutritional imbalance disrupts this ecosystem and its integration with the host remains poorly understood. Using an isocaloric but nutrient-imbalanced diet in a mouse model, we recapitulated chronic growth stunting and identified a systemic syndrome comprising growth retardation, oxidative stress, and immunosuppression. Gut microbiota dysbiosis was established as the central mediator of these pathological manifestations. Integrated multi-omics analyses revealed that nutritional imbalance compromised microbial community structure and function, reducing diversity and ecological stability whereas disrupting metabolic activity—particularly short-chain fatty acid production. Through single-cell ribonucleic acid sequencing of colonic epithelium, we demonstrated that these microbial perturbations suppressed host energy metabolism and inhibited the Wnt/β-catenin signaling pathway in regenerative epithelial cells. This resulted in the downregulation of key stem cell regulators LGR5 and ASCL2, ultimately impairing epithelial renewal and manifesting as reduced crypt depth. Our findings reveal a microbiota-dependent mechanism linking dietary imbalance to impaired host development, demonstrating how nutritional stress disrupts gut microbial structure and metabolic output, ultimately compromising epithelial regeneration. This work highlights the importance of considering the gut microbiome as an ecosystem whose homeostasis is fundamental to early-life health.

## Introduction

Children and adolescents represent the cornerstone of our collective future. However, a substantial number of children continue to suffer from malnutrition, which poses a serious threat to their survival and healthy development [1, 2]. According to the latest joint estimates by the UNICEF–WHO–World Bank Group (2024), ~150 million children under 5 years of age were affected by stunting, and as many as 42.8 million were suffering from wasting worldwide. Should current trends persist, tens of millions of children will remain left behind by 2030, jeopardizing the achievement of the relevant United Nations Sustainable Development Goals.

It is critical to emphasize that malnutrition is not synonymous with energy deficiency; it encompasses diverse conditions beyond undernutrition, including stunting and obesity [3, 4]. These conditions might stem from imbalances in nutrient intake rather than inadequate energy intake. Advances in staple crop production have shifted the primary nutritional challenge for many children from a lack of sufficient food to a lack of adequate dietary quality [5, 6]. Therefore, the focus is now shifting to chronic childhood stunting driven by imbalances in dietary structure and quality, including lack of diversity [7], inadequate protein quality [8, 9], excessive refined carbohydrates with deficits in fatty acids and dietary fiber [10, 11]. However, the pathophysiological landscape of stunting induced by dietary imbalances, including its manifestations in organ development, metabolic homeostasis, and tissue growth, has yet to be comprehensively mapped, leaving the underlying mechanisms poorly defined.

Evidence indicates that the early-life colonization and assembly of the gut microbiota can program the host’s physiology with long-term, and potentially lifelong, consequences [12–14]. Dietary patterns are known to rapidly and significantly reshape the ecology and metabolic functions of the gut microbial community. For example, dietary fiber deprivation promotes gut microbiota to degrade the mucus layer, leading to its erosion, barrier thinning, and increased susceptibility to pathogens [15], whereas a high-protein diet elevates succinate production by the gut microbiota, which in turn drives the generation of bacterial reactive oxygen species and extracellular vesicles [16]. However, how the gut microbiota responds to nutritional imbalances in diet and how such a dysbiosis community in turn negatively regulates host growth and development remain to be elucidated.

To model malnutrition, we formulated a nutrient-imbalanced, isocaloric diet characterized by an imbalanced macronutrient profile (extremely high carbohydrate, low protein/fat content). The observed phenotypic divergence necessitated a multi-omics investigation. We first utilized 16S ribosomal ribonucleic acid (rRNA) gene sequencing to delineate the diet-induced perturbations in gut microbiota structure, maturity, and stability. This was complemented by metagenomic and targeted metabolomic analyses to uncover the associated functional metabolic shifts within the gut environment. Finally, single-cell transcriptomics and biochemical assays were employed to interrogate the dynamic response of the host gut epithelium.

## Materials and methods

### Animals and experimental design

A total of 30 3-week-old female specific pathogen-free (SPF) C57BL/6 J mice (Sibeifu Biotechnology Co., Ltd., China) were used in the experiment and underwent a 7-day acclimation period before treatment. All mice were housed in an SPF animal barrier facility with room temperature (23.0 ± 2.0°C), relative humidity 50%–60%, and a 12-h light/dark cycle. To control for the substantial inter-individual variability in baseline microbiota and to establish a standardized starting point for assessing the sole impact of diet, we initially depleted the indigenous gut microbiota using a broad-spectrum antibiotic cocktail. The mice were then treated for 7 days with an antibiotic mixture (ampicillin 1 g/L, neomycin 1 g/L, metronidazole 1 g/L, vancomycin 0.5 g/L, diluted in ultrapure water) to eliminate the gut microbiota. Specifically, mice had unrestricted access to the antibiotic water and additionally received a daily gavage of 200 μL of the antibiotic mixture. During both the acclimation and antibiotic treatment periods, mice were fed a normal diet (AIN-93G, Table S1). During the formal experimental period, the 30 mice were randomly divided into two groups (*n* = 15; 5 mice/cage; CON and MAL). The mice in each group were fed corresponding customized diets for 5 weeks (Table S1). Detailed composition and nutrient profiles of the CON and MAL diets are provided in Table S1. Both diets were formulated to be isocaloric, with the MAL diet characterized by higher carbohydrate content (primarily from starch) and reduced levels of protein (casein), fat (soybean oil), and fiber (cellulose) compared to the CON diet. Nutritionally, compared to the CON diet, the MAL diet contained higher carbohydrates and lower protein and fat. In terms of ingredients, the MAL diet contained more starch, whereas the CON diet contained higher levels of casein, cellulose, and soybean oil. It is important to emphasize that the two diets were isocaloric. Body weight of each mouse was measured daily and fecal samples were collected weekly from all mice. After 35 days, all animals were euthanized, with blood, liver, and colonic tissue samples collected for subsequent analysis.

### The analysis of serum samples

Serum was used to analyze several parameters including immunity indicators [immunoglobulin A (IgA), immunoglobulin G (IgG), and immunoglobulin M (IgM)], antioxidant index [total superoxide dismutase (T-SOD), total antioxidant capacity (T-AOC), glutathione peroxidase (GSH-PX), malondialdehyde (MDA), and glutathione (GSH)], inflammatory factors [interleukin-1β (IL-1β), interleukin-6 (IL-6), and tumor necrosis factor-α (TNF-α)], and insulin-like growth factor-1 (IGF-1). The concentrations of IgG, IL-1β, IL-6, and TNF-α were measured with kits from Beijing Laibo Terui Technology Development Co., Ltd. (Beijing, China). The concentrations of T-SOD, T-AOC, GSH-PX, and MDA were determined using an automated biochemistry analyzer (model 7600, Hitachi High-Tech Scientific Solutions Co., Ltd., Kyoto, Japan). The IGF-1 was detected using ELISA kits in an enzyme-immunoassay instrument (Multiskan MK3, ThermoFisher Scientific Co., Ltd., Waltham, MA, USA).

### Histomorphologic examinations

Colonic tissue samples were dehydrated through a graded ethanol series, embedded in paraffin, and sectioned at 6 μm thickness. Sections were stained with hematoxylin and eosin, and colonic structures were examined under an Olympus BX-51 light microscope (Olympus Corporation, Tokyo, Japan) at 20× magnification. Microscopic evaluation was blindly performed by an experienced pathologist.

### Deoxyribonucleic acid extraction, polymerase chain reaction amplification, and data processing

A total of 150 fecal samples of mice were collected across the whole experiment. Microbial deoxyribonucleic acid (DNA) was extracted from fecal samples of mice using the Dneasy PowerLyzer PowerSoil Kit (Qiagen, Hilden, Germany). The quality of the extracted DNA was assessed with a Thermo NanoDrop 2000 UV-microspectrophotometer and by 1% agarose gel electrophoresis. The V3–V4 hypervariable regions of the bacterial 16S rRNA gene were amplified using the primer pair 338F (5′-ACTCCTACGGGAGGCAGCAG-3′) and 806R (5′-GGACTACHVGGGTWTCTAAT-3′) on an ABI GeneAmp 9700 polymerase chain reaction (PCR) thermocycler (Applied Biosystems, CA, USA). PCR products were visualized on 2% agarose gels, excised, and purified using the AxyPrep DNA Gel Extraction Kit (Axygen Biosciences, Union City, CA, USA) according to the manufacturer’s instructions, followed by quantification with the Quantus Fluorometer (Promega, USA). The resulting amplicon libraries were sequenced on a MiSeq System platform (Illumina, San Diego, CA, USA; PE250).

Raw FASTQ files were demultiplexed using in-house Perl scripts, followed by quality filtering with fastp (version 0.19.6) and merging with FLASH (version 1.2.11) [17]. Unique amplicon sequence variants (ASVs) were inferred using the DADA2 pipeline. Taxonomic assignment of ASVs was performed in QIIME2 with a naive Bayes consensus classifier trained on the SILVA 16S rRNA database (version 138), and ASV abundances were subsequently adjusted based on predicted rRNA operon copy numbers derived from the rrnDB database [18].

### Absolute quantification of total bacteria and *Bifidobacterium* by quantitative real-time PCR

Universal bacterial primers targeting the 16S rRNA gene (F: 5′-ACTCCTACGGGAGGCAGCAG-3′; R: 5′-ATTACCGCGGCTGCTGG-3′) were used for total bacteria quantification, whereas *Bifidobacterium*-specific primers (F: 5′-CGCGTCCGGTGTGAAAG-3′; R: 5′-CTTCCCGATATCTACAC-ATTCCA-3′) were used for genus-specific quantification. All reactions were performed in triplicate using TB Green Premix Ex Taq II (Takara, Japan) on a Roche LightCycler 480II system (Roche, Germany). Thermal cycling conditions were: 95°C for 5 min, followed by 40 cycles of 95°C for 10 s and 60°C for 30 s. Melting curve analysis was performed to verify amplification specificity. Standard curves and absolute quantification: For total bacteria, standard curves were generated using 10-fold serial dilutions of purified PCR products with known copy numbers (10^4^ to 10^8^ copies/μL). For *Bifidobacterium*, plasmid standards containing the target gene sequence were constructed and serially diluted (10^1^ to 10^6^ copies/μL). Copy numbers in fecal samples were calculated based on Ct values and the corresponding standard curves (*R*^2^ > 0.99). The absolute abundance was expressed as copies per microliter of DNA extract.

### Metagenomic sequencing and processing

A total of 30 fecal samples of mice at the end of the experiment were selected to perform the metagenomic shotgun sequencing. Genomic DNA was sheared to an average size of ~400 bp using a Covaris M220 focused-ultrasonicator (Gene Company Limited, Shanghai, China). Paired-end sequencing libraries were constructed with the NEXTFLEX Rapid DNA-Seq Kit (Bioo Scientific, Austin, TX, USA). Sequencing was performed on a NovaSeq 6000 System (Illumina, San Diego, CA, USA.) using NovaSeq 6000 S4 reagent kits according to the manufacturer’s instructions at www.illumina.com. Raw sequencing reads were processed with fastp (version 0.20.0; https://github.com/OpenGene/fastp) to remove adapter sequences and low-quality reads (length < 50 bp, quality score < 20, or containing N bases). To exclude host-derived sequences, reads were aligned to the bovine reference genome, and any reads or their mates mapping to the host genome were discarded. Quality-filtered data were assembled using MEGAHIT (version 1.1.2; https://github.com/voutcn/megahit), and contigs with length ≥ 300 bp were retained as the final assembly. Open reading frames (ORFs) were predicted from the assembled contigs using Prodigal (version 2.6.3; https://github.com/hyattpd/Prodigal), and ORFs ≥100 bp in length were retained. A non-redundant gene catalog was constructed using CD-HIT (version 4.7; http://weizhongli-lab.org/cd-hit/) with thresholds of 90% sequence identity and 90% coverage. Gene abundances in each sample were estimated using SOAPaligner (version 2.21; https://github.com/ShujiaHuang/SOAPaligner) with a 95% identity threshold. Taxonomic annotation of non-redundant genes was performed by aligning them to the NCBI NR database using DIAMOND (v2.0.11; http://ab.inf.uni-tuebingen.de/software/diamond/) with an e-value cutoff of 1e^−5^. Functional annotation of non-redundant genes was similarly conducted against the KEGG databases [19, 20].

### Determination of fecal fermentation parameters

Fecal samples were thawed at 4°C and subsequently centrifuged at 2500 × g at room temperature. Subsequently, 1 ml of supernatant from each sample was separated and transferred into a 1.5 ml microcentrifuge tube containing 0.2 ml of metaphosphoric acid solution (25% w/v). The mixture was then placed in a water bath, followed by centrifugation at 10 000 × g for 30 min at 4°C. The resulting supernatant was collected and stored at 4°C for subsequent analysis. Volatile fatty acid (VFA) concentrations were determined using gas chromatography (GC-6800, Beijing Beifen Tianpu Instrument Technology Co., Ltd., China) [21].

### Single-cell ribonucleic acid sequencing of colon and data processing

For single-cell RNA sequencing (scRNA-seq), three biological replicates were randomly selected from each of the CON and MAL groups. The DNBelab C4 system utilizes an in-house developed multi-bead recognition technology based on Drop-seq. Each magnetic bead is functionally modified with an average of 10^7^ capture sequences of two types: long sequences for mRNA capture, and short sequences designed to bind mRNA released from small-sized magnetic beads. Within each droplet, small-sized beads integrate capture information from multiple large-sized beads, thereby enhancing the experimental system and increasing cell capture efficiency.

A 0.4% trypan blue staining was applied to assess nuclei/cell viability under microscopy, and samples with >80% viability were processed for library construction. Single-cell RNA-seq libraries were prepared using the DNBelab C Series High-throughput Single-Cell RNA Library Preparation Set V3.0 (MGI, China). Single-cell suspension, oil, and beads were sequentially loaded into the C4 scRNA slide and instrument. Droplets were subsequently broken using a vacuum pump to release mRNA–bead complexes, followed by complementary DNA (cDNA) synthesis under optimized thermal conditions. The resulting cDNA and Oligo products were amplified and purified, then assessed for concentration and size distribution to ensure quality specifications were met.

For oligo library construction, products underwent amplification, index ligation, and purification prior to circularization. cDNA was fragmented, end-repaired, and A-tailed before adapter ligation under defined conditions. Following purification, adapter-ligated cDNA was amplified by PCR and again purified. Both cDNA and Oligo products were denatured into single strands, and circularization was performed under programmed conditions. Single-stranded circular products were obtained, and any residual linear DNA was digested. Using rolling circle amplification, single-stranded circular DNA was replicated to generate DNA nanoballs (DNBs), each containing multiple copies of the original library molecules. High-quality DNBs were loaded into patterned nanoarrays and sequenced by combinatorial Probe-Anchor Synthesis. The cDNA library was sequenced as PE 47 + 100 and the Oligo library as PE 32 + 42.

The raw gene expression matrix was generated from sequencing data using the DNBelab_C4scRNA pipeline (version 1.0.1, https://github.com/MGI-tech-bioinformatics/DNBelab_C_Series_scRNA-analysis-software) [22]. Downstream analyses were performed with the Seurat R package (version 3.2.0, https://satijalab.org/seurat/) [23]. Quality control filtering was applied based on two metrics: cells with <200 detected genes or those representing the upper 0.1% of gene counts were excluded; additionally, cells within the top 15% of mitochondrial read ratios were removed. Potential doublets were identified and eliminated using DoubletDetection (https://rdrr.io/github/scfurl/m3addon/man/doubletdetection.html). Cell cycle phase assignment was performed using the CellCycleScoring function in Seurat. The gene expression dataset was normalized and subjected to principal component analysis (PCA; *n* = 15) using the top 2000 highly variable genes. Dimensionality reduction and two-dimensional visualization were conducted using UMAP. Cluster-specific marker genes were identified with the FindAllMarkers function in Seurat (logfc.threshold >0.25, min.pct > 0.1, and adjusted *P* value ≤.05). Cell type annotation was performed using the SCSA method (https://github.com/bioinfo-ibms-pumc/SCSA) [24]. Differential expression analysis between sample groups was carried out with the FindMarkers function under the same statistical thresholds. Kyoto Encyclopedia of Genes and Genomes (KEGG, version 93.0) pathway enrichment analysis was performed using the phyper function in R, with pathways showing false discovery rate ≤ 0.05 considered significantly enriched.

### Statistics

One-way analysis of variance (ANOVA) in SPSS 19.0 (SPSS Inc., Chicago, IL, USA) was used to compare the differences of phenotypic parameters and blood indexes. A two-tailed Wilcoxon signed-rank test was applied to compare group differences in alpha diversity metrics. For beta diversity, analysis of similarity (ANOSIM) was employed to test for significant separation based on Bray–Curtis distance matrices.

A random forest regression model was implemented to identify age-associated bacterial taxa in mouse feces using the randomForest R package (version 4.6-14). Model parameters were configured with “importance” and “proximity” set to TRUE, and “ntree” set to 10 000 to ensure robust feature selection and model stability.

Spearman’s analysis was performed to calculate the correlations of bacteria at the different growth stages using “psych” package in R, and the related interaction networks were visualized using the Gephi (https://gephi.org/). To remove random noise arising from finite sample sizes in the correlation matrix while retaining genuine ecological co-occurrence relationships, we employed a Random Matrix Theory (RMT)-based approach to determine the optimal correlation threshold. For each network, we traversed correlation coefficient thresholds *r* from 0.30 to 0.80 in 26 equally spaced steps. At each threshold, the corresponding correlation matrix was retained (binary adjacency matrix), and its eigenvalue spectrum was analyzed. Spline unfolding was performed to obtain the Nearest-Neighbor Spacing Distribution (NNSD) of the eigenvalues. For a purely random correlation matrix, the NNSD follows the Gaussian Orthogonal Ensemble (GOE) distribution (Wigner distribution). As non-random, biologically meaningful interactions are retained, the NNSD gradually transitions toward a Poisson distribution. We quantified the agreement between the observed NNSD and the Poisson distribution using the Kolmogorov–Smirnov (KS) test. The threshold *r* at which the KS *P* value first exceeded .05 was selected as the final cutoff r. If no threshold satisfied this criterion, we instead selected the *r* value that minimized the sum of squared residuals (SSE) of the exponential fit (Poisson) as r. This procedure was applied independently to each network (each time point and each dietary group). The resulting *r* values were all <0.68. Therefore, a uniform threshold of *r* = 0.70 (which is strictly greater than the maximum *r* across all networks) was adopted for constructing all co-occurrence networks to ensure both statistical non-randomness and cross-group comparability. Therefore, only significant connections (*P* < .05, |*r*| > 0.70) were shown in the networks. The degree of nodes was applied to represent the network sparsity and the lower the degree, the sparser the network is [25]. To further assess network robustness, we calculated the average node degree and the natural connectivity, the latter being an eigenvalue-based metric that quantifies a network’s resilience to node removal by summing exponential functions of the eigenvalues of the adjacency matrix. Compared with conventional indices (degree or betweenness), natural connectivity is more sensitive to subtle topological changes and has been widely used to characterize the stability of microbial co-occurrence networks [26]. Line plots were illustrated using the ggplot2 package in R.

## Results

### Malnutrition impairs systemic metabolism and colon development in mice

**Figure 1:**
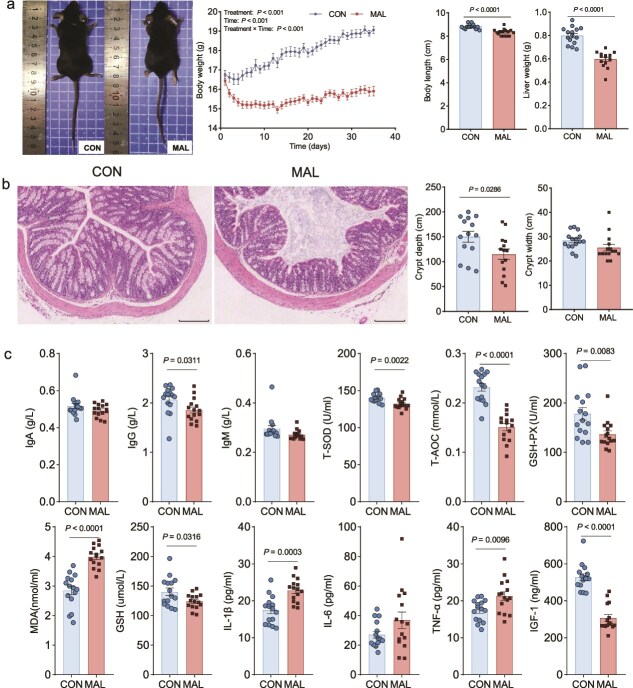
Effects of MAL treatment on growth performance, colonic morphology and blood parameters. (a) Growth performance parameter (body condition, body length, liver weight) and temporal dynamics of body weight in CON and MAL groups. (b) The difference of colonic morphological indices (crypt width, crypt depth) between the CON and MAL groups. The scale bar indicates a length of 200 μm. (c) Antioxidant capacity (T-AOC, GSH-PX, MDA, and GSH), immune function (IgA, IgG, and IgM), and inflammatory response (IL-1β, IL-6, and TNF-α) indices, as well as IGF-1 level, in the CON and MAL groups. Statistical significance between the two groups was determined at *P* < .05.

We first assessed the effects of malnutrition on mouse growth phenotypes by examining a comprehensive set of physiological parameters, from organ development and gut architecture to systemic markers of oxidative stress, inflammation, and immune status. As a result, body weight (top-left) showed significant differences between CON and MAL groups over 0–40 days, with two-way ANOVA confirming main effects of Treatment (*P* < .001), Time (*P* < .001), and their interaction (*P* < .001). Mice in the MAL group had lower body weight across the whole experiment. Body length (top-middle) and liver weight (top-right) were also significantly reduced in the MAL group (*P* < .0001), indicating stunted growth and potential hepatic impairment (Fig. 1a). Although we did not observe the significant differences in colon length (Fig. S1), MAL treatment significantly decreased colonic crypt depth compared to the CON group (*P* = .0286). Crypt width trended lower but did not meet explicit significance, suggesting possible gut structural damage and compromised barrier function (Fig. 1b). In addition, we next turned to the systemic physiological effects, evaluating the impact of malnutrition on serum biochemical profiles in mice (Fig. 1c). MAL diet reduced T-AOC (*P* < .0001), GSH-PX (*P* = .0083), and T-SOD (*P* = .0022). In contrast, MDA increased (*P* < .0001), and GSH decreased (*P* < .0001), showing severe oxidative stress in MAL-treated mice. In terms of inflammation and growth factor, MAL elevated TNF-α (*P* = .0316), IL-1β (*P* = .0096), and IL-6 (*P* = .0003). IGF-1 was reduced (*P* = .0096), suggesting a potential link between inflammation and impaired growth. We observed IgG was lower in the MAL group (*P* = .0311). IgM and IgG trended downward, indicating suppressed immunity, which may worsen systemic stress. Finally, to visually encapsulate the global shift in serum profile, we performed PCA on all serum parameters. This analysis revealed a clear separation between CON and MAL groups (*r* = 0.85, *P* = .001; Fig. S2), confirming the coordinated systemic response to nutritional imbalance.

### Malnutrition induces gut microbiota dysbiosis in mice

**Figure 2:**
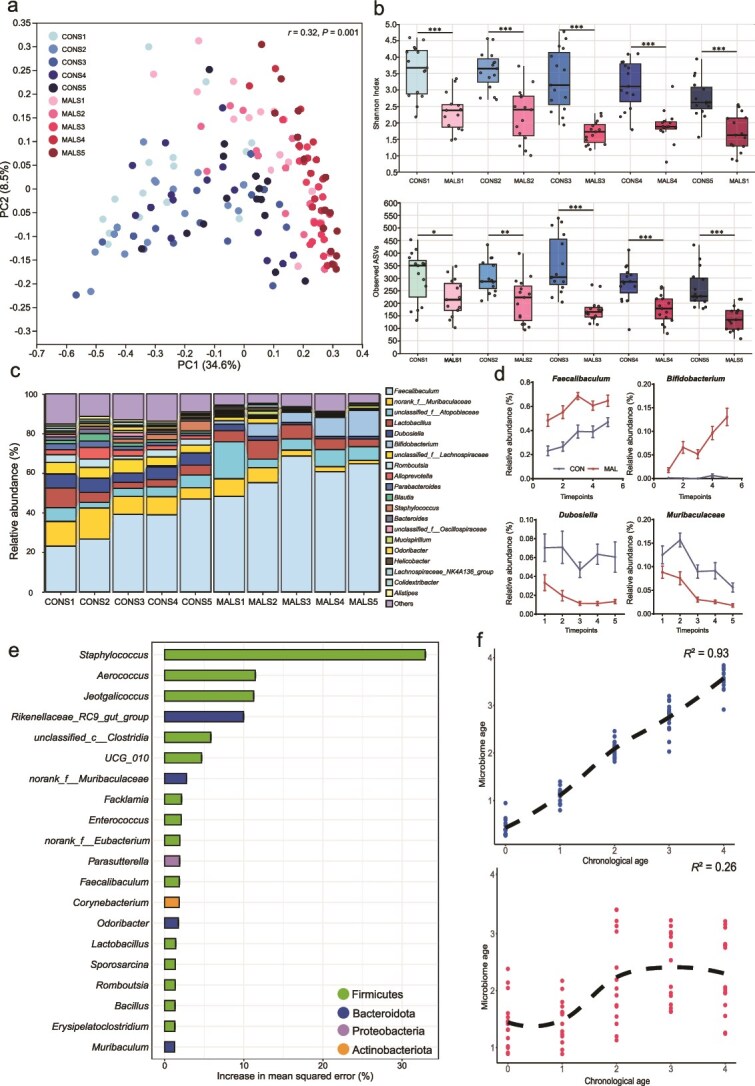
MAL treatment-induced alterations in gut microbiome composition, structure and age-related characteristics in experimental subjects. (a) PCoA of gut microbial community structure in CON (CONS1 to CONS5) and MAL-treated (MALS1 to MALS5) groups. (b) The changes in number of observed ASVs and Shannon index (ASV level) of gut microbiota with growth stages in the CON and MAL groups. Only the significant correlations were marked (^*^P < .05, ^**^0.01 < *P* < .05, ^***^P < .01). (c) Temporal dynamics of gut microbial genus relative abundance in the CON and MAL groups across different timepoints. (d) Comparison of key gut microbial genus relative abundance (such as *Faecalibaculum, Bifidobacterium*, and *Dubosiella*) between the CON and MAL groups. (e) The top 20 age-related signature microbiota of the gut in mice were listed based on the mean square error of predictions (%IncMSE). (f) The distinct capability for age prediction from gut microbiota of mice in the CON and MAL groups. Statistical significance between the two groups was determined at *P* < .05.

The gut microbiome serves as a key mediator of host physiological homeostasis, particularly in gut tissue development. Given that dietary differences represent a critical driver of gut microbiota modulation, it is reasonable to hypothesize that gut dysbiosis induced by the MAL diet contributes to chronic malnutrition in mice. Therefore, we employed 16S rRNA gene sequencing to investigate characteristic indices of the mouse gut microbiota, including community structure, diversity, maturity, and stability, aiming to evaluate the extent of adverse effects exerted by MAL diet. To establish a baseline reference prior to dietary intervention, we first compared the gut microbial communities of CON and MAL groups from fecal samples collected immediately before the start of the experimental diets. Principal coordinates analysis (PCoA) based on Bray–Curtis dissimilarities revealed no significant separation between the two groups at baseline (ANOSIM, *r* = 0.007, *P* = .533; Fig. S3), confirming that the groups started with comparable microbial compositions. This baseline similarity ensures that subsequent differences observed during the experimental period can be reliably attributed to the dietary interventions. According to the principal coordinate analysis (PCoA) result (Fig. 2a), there was a distinct separation between the two groups across all the 5 timepoints, which was supported by the ANOSIM analysis (*r* = 0.3239, *P* = .001). The result confirmed that MAL drove reproducible restructuring of the gut microbial assemblage. Similarly, the Shannon Index and number of observed ASVs revealed a significant decline in microbial diversity and richness in the MAL group respectively (Fig. 2b). Specifically, compared with the CON diet, the MAL diet led to a marked reduction in gut microbial complexity that persisted throughout the study (*P* < .05). For the microbial composition, at the phylum level, *Firmicutes* constituted the dominant bacteria in the both groups, followed by *Bacteroidota* and *Actinobacteriota* (Fig. S4). We observed a significant difference in taxonomic composition at the genus level between the two groups (Fig. 2c). Although *Faecalibaculum* remained the dominant genus in both groups and increased over time, its abundance was significantly and persistently higher in the MAL group than in the controls throughout the study, which was also similar with *Bifidobacterium*. In contrast, *norank_f__Muribaculaceae, Dubosiella*, and *unclassified_f__Lachnospiraceae* were significantly enriched in the CON group compared with the MAL group (Fig. 2d). To validate that the observed microbial changes reflect genuine abundance differences rather than artifacts arising from relative abundance measurements, we performed absolute quantification of total bacteria and *Bifidobacterium* by qPCR across all five experimental stages. Total bacterial abundance did not differ significantly between CON and MAL groups at any time point (Fig. S5a), confirming that antibiotic pre-treatment successfully standardized baseline microbial load. Consistent with the relative abundance data, the absolute abundance of *Bifidobacterium* was significantly higher in the MAL group compared to the CON group at all time points (*P* < .001; Fig. S5b), validating that the observed differences represent true changes in bacterial abundance. Using a random forest algorithm, we identified age-associated microbial taxa in CON mice including *Staphylococcus, Aerococcus, Jeotgalicoccus*, and *Rikenellaceae_RC9_gut_group* (Fig. 2e), and constructed a microbiota maturation curve, which was strongly correlated with host chronological age (*R*^2^ = 0.93). In contrast, the MAL group exhibited a significant lag in this microbial maturation trajectory (*R*^2^ = 0.26), indicating that dietary intervention disrupted the normal progression of gut community assembly (Fig. 2f).

**Figure 3:**
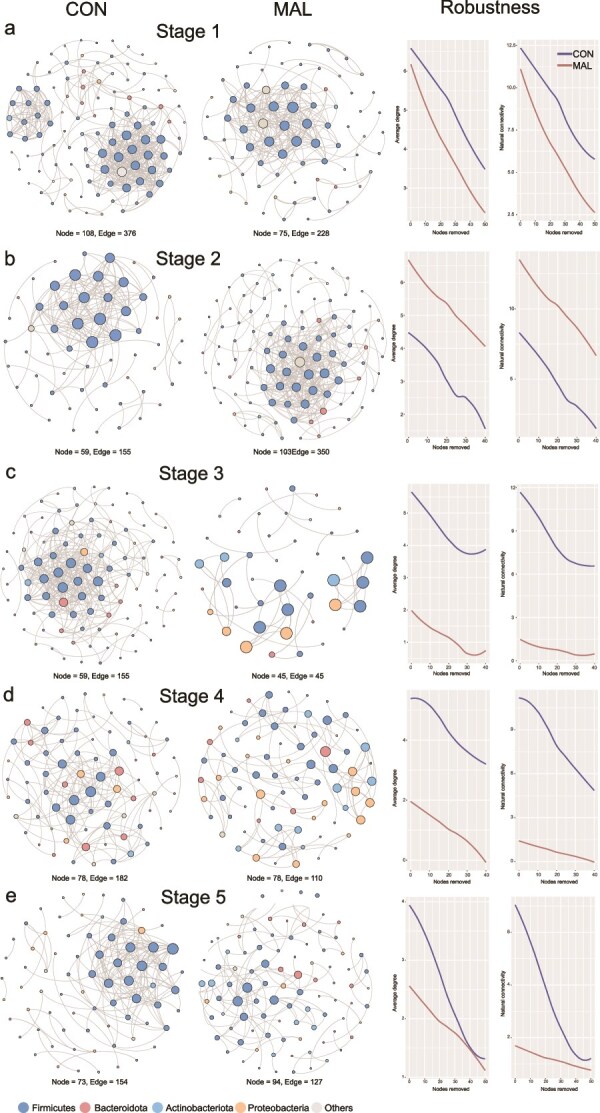
MAL treatment modulates gut microbial co-occurrence network properties across different experimental stages. (a) The microbial co-occurrence network properties in the two groups at the Stage 1. (b) The microbial co-occurrence network properties in the two groups at the Stage 2. (c) The microbial co-occurrence network properties in the two groups at the Stage 3. (d) The microbial co-occurrence network properties in the two groups at the Stage 4. (e) The microbial co-occurrence network properties in the two groups at the Stage 5.

To determine an objective correlation threshold for network construction, we applied RMT method to each microbial co-occurrence network across all time points of the two groups. The RMT analysis identified the critical threshold *r* (the minimum correlation coefficient at which the NNSD deviates from the random matrix prediction) for each network (Figs S6 and S7). The maximum *r* across all networks was 0.68. Therefore, we selected a uniform threshold of *r* = 0.70 for constructing all co-occurrence networks, which is strictly greater than every network-specific *r* and ensures both statistical non-randomness and cross-group comparability. To further dissect the impact of MAL treatment on gut microbial community structure, we analyzed the robustness of microbial co-occurrence networks across CON and MAL groups at distinct stages. Network robustness, assessed by simulating random node removal, reflects the structural tolerance of microbial communities. For network robustness, with the exception of Stage 2 (Fig. 3b), Stage 1 networks in the CON group exhibited higher structural complexity, characterized by 108 nodes and 376 edges, compared to the MAL group (75 nodes, 228 edges) (Fig. 3a), this trend persisted through subsequent stages, with Stage 3 CON networks retaining more nodes (59 vs. 45) and edges (155 vs. 45) than MAL networks (Fig. 3c), and Stage 4 CON networks maintaining more edges (182 vs. 110) despite identical node counts (Fig. 3d). Similarly, Stage 5 network also showed a more complex connections in the CON group compared with the MAL group (154 edges vs. 127 edges) (Fig. 3e). When assessing resistance to node removal (a metric of robustness), the CON group showed a slower decline in network integrity with increasing node removal (40–50 nodes removed) relative to the MAL group (Fig. 3a, c, d, and  e), indicating enhanced resilience of CON microbial networks to perturbation. Complementary analysis of natural connectivity—another critical index of network stability reflecting the ability to maintain communication between nodes—revealed consistent reductions in the MAL group across stages, further confirming that MAL impaired the structural stability of gut microbial co-occurrence networks. At the phylum level, the CON group maintained a more balanced distribution of dominant phyla (*Firmicutes, Bacteroidota, Actinobacteriota, Proteobacteria*) and “Others”, whereas the MAL group exhibited disrupted phylum-level abundance patterns, which likely underpin the observed network instability. To delineate the functional metabolic consequences of the MAL intervention, we performed integrated metagenomic and targeted metabolomic profiling.

### Malnutrition alters gut microbial metabolic function in mice

**Figure 4:**
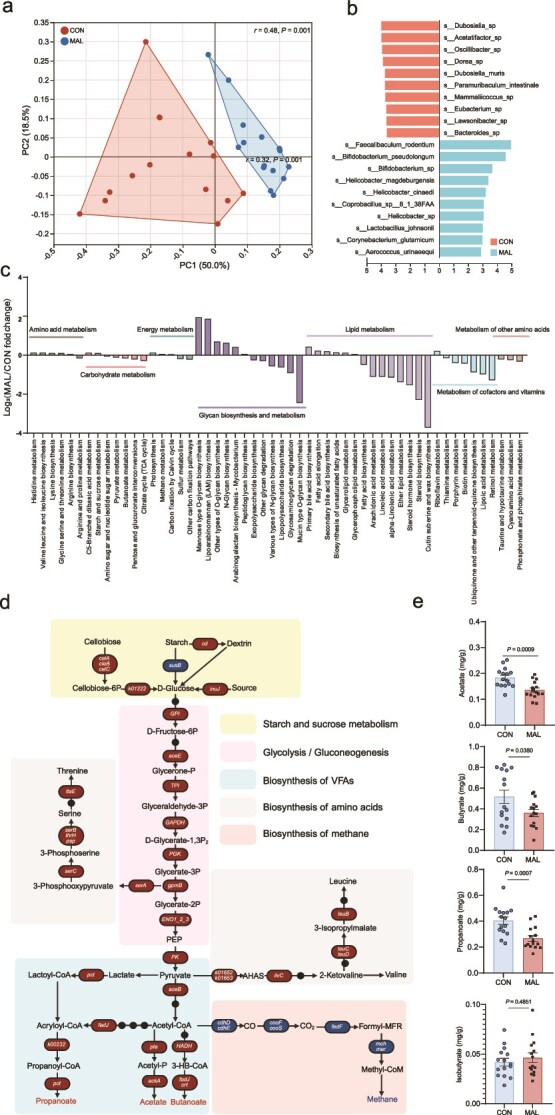
MAL treatment reshapes gut microbial species composition, functional pathways, and metabolic pattern in mice. (a) PCoA of gut microbial community structure in the CON and MAL group. (b) The signature species in the CON and MAL groups identified by linear discriminant analysis effect size (LEfSe) analysis. (c) The difference of gut microbial metabolic functions in the CON and MAL groups. (d) Schematic representation of key metabolic pathways affected by MAL treatment. (e) Comparison of SCFA concentrations between CON and MAL group. Statistical significance between the two groups was determined at *P* < .05. *celA*: cellulase A; *celB*: cellulase B; *celC*: cellulase C; *k01222*: 6-phospho-beta-glucosidaseD-Glucosidase; *susB*: glucan 1,4-alpha-glucosidase; *cd*: glucan 1,4-alpha-glucosidase; inuJ: inulosucrase; *ltaE*: threonine aldolase; *serB, thrH, psp*: phosphoserine phosphatase; *serC*: phosphoserine aminotransferase; *serA*: D-3-phosphoglycerate dehydrogenase/2-oxoglutarate reductase; *GPI*: glucose-6-phosphate isomerase; *aceE*: pyruvate dehydrogenase E1 component; *TPI*: triosephosphate isomerase; *GAPDH*: glyceraldehyde 3-phosphate dehydrogenase; *PGK*: phosphoglycerate kinase; *gpmB*: phosphoglycerate kinase; *ENO1_2_3*: enolase 1/2/3; *k00232*: acyl-CoAoxidase; *pct*: propionate CoA-transferase; *fadJ*: 3-hydroxyacyl-CoA dehydrogenase/enoyl-CoA hydratase/3-hydroxybutyryl-; *PK*: pyruvate kinase; *aceB*: malate synthase; *pta*: phosphate acetyltransferase; *ackA*: acetate kinase; *HADH*: 3-hydroxyacyl-CoA dehydrogenase; *fadJ*:3-hydroxyacyl-CoA dehydrogenase/enoyl-CoA hydratase/3-hydroxybutyryl-CoA epimerase; *crt*: short-chain-enoyl-CoA hydratase; *k01652*: acetolactate synthase I/II/III large subunit; *k01653*: acetolactate synthase I/III small subunit; *ilvC*: ketol-acid reductoisomerase; *leuB*: 3-isopropylmalate dehydrogenase; *leuC*: 3-isopropylmalate/(R)-2-methylmalate dehydratase large subunit; *leuD*: 3-isopropylmalate/(R)-2-methylmalate dehydratase small subunit; *cdhD, cdhE*: acetyl-CoA decarbonylase/ synthase; *cooF*: anaerobic carbon-monoxide dehydrogenase iron sulfur subunit; *cooS*: anaerobic carbon-monoxide dehydrogenase catalytic subunit; *fedF*: ferredoxin; *mch*: methenyltetrahydromethanopterin cyclohydrolase; *mer*: 5,10-methylenetetrahydromethanopterin reductase;

Consistent with the 16S rRNA gene sequencing results, species-level taxonomic assignment confirmed that the MAL diet significantly reduced gut microbial diversity (Fig. S8) and altered community composition (Fig. 4a). Specifically, taxa including *Dubosiella* sp*., Acetatifactor* sp*.*, and *Oscillibacter* sp*.* were identified as signature bacteria of the CON group, where they were highly enriched (*P* < .05) (Fig. 4b). In contrast, the MAL group was characterized by a distinct set of indicator species *Faecalibaculum rodentium, Bifidobacterium pseudolongum*, and *Helicobacter magdeburgensis* (*P* < .05). Microbial function results revealed that the MAL intervention broadly suppressed the microbial capacity for nutrient metabolism. In contrast to the CON group, the MAL group exhibited marked reductions in functional pathways related to the metabolism of carbohydrates, lipids, and amino acids, as well as in biosynthetic pathways for cofactors and vitamins (Fig. 4c). In addition, we further identified the main microbial taxa driving the top 10 most abundant functional pathways. Whereas *F. rodentium* and *Granulimonas faecalis* served as core contributors in both groups, several taxa were uniquely associated with each diet: *Olsenella* sp*. CU969, Dubosiella* sp*.*, and *Dubosiella muris* were specific to the MAL group, whereas *B. pseudolongum* was exclusive to the CON group (Fig. S9). A total of 4653 differentially abundant genes were identified between the two groups (1083 genes enriched in the CON group and 3570 enriched in the MAL group) (*P* < .05) Then, pathway map (Fig. 4d) of enrichment analysis illustrated that MAL treatment impaired the flow of key carbon substrates through carbohydrate metabolism: it disrupted starch and sucrose degradation (blocking the breakdown of cellobiose, dextrin, and starch into D-glucose and D-fructose-6P) and glycolysis/gluconeogenesis (reducing the conversion of glyceraldehyde-3P to pyruvate via downregulated of enzyme genes like GAPDH and PGK), which directly limited the production of pyruvate—an essential precursor for short-chain fatty acid (SCFA) biosynthesis. This upstream impairment cascaded to SCFA metabolism, with reduced flux through acetate, propanoate, and butanoate biosynthesis pathways (evidenced by suppressed activity of genes like pct for propanoate and *fadJ* for butanoate) and was accompanied by disruptions in amino acid metabolism (e.g. reduced conversion of 3-phosphoserine to serine, and 3-phosphooxypyruvate to valine/leucine via downregulated *leuC, leuD*, and *ilvC*). In contrast, non-beneficial pathways like methane biosynthesis exhibited relative upregulation, reflecting a maladaptive shift in microbial metabolic output. Together, these results confirmed that MAL treatment did not merely alter microbial taxonomic composition but induced a systemic change of gut microbial metabolism. Finally, we quantified the concentrations of major SCFAs in the gut of mice (Fig. 4e). Consistent with the inferred metabolic pathway, the levels of acetate, propionate, and butyrate as key end products of microbial fermentation were lower in the MAL group than that in the CON group (*P* < .05).

### Colonic epithelial energy metabolism and proliferation are regulated by bacterial metabolites

To link gut microbial functional perturbations induced by MAL treatment to host gut cellular responses, we performed scRNA-seq to characterize cell type composition, metabolic pathway activity, and responses to microbial metabolites in the colonic tissue of CON and MAL groups.

**Figure 5:**
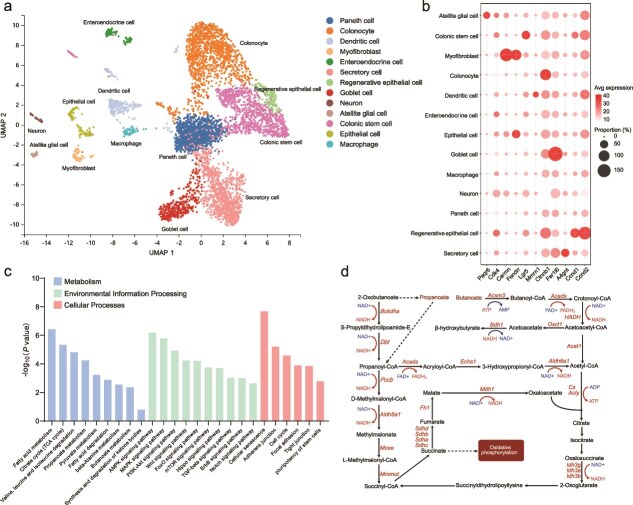
Construction of the single-cell landscape of the colonic cells from mice in the CON and MAL groups. (a) UMAP visualization of colonic single-cell populations in the CON and MAL groups. (b) The identification of marker genes in the different cell populations. (c) Functional enrichment analysis of differentially expressed genes in the regenerative epithelial cells. Top 3 enriched pathways for each category: metabolism—fatty acid metabolism, citrate cycle (TCA cycle), valine/leucine/isoleucine degradation, propanoate metabolism and pyruvate metabolism; environmental information processing—AMPK signaling pathway, MAPK signaling pathway, PI3K-Akt signaling pathway and Wnt signaling pathway; cellular processes—cellular senescence, adheres junction, and cell cycle. (d) The difference of schematic of critical metabolic pathways in the regenerative epithelial cells of colon. *Bckdha*: branched chain keto acid dehydrogenase E1 subunit alpha; *Dbt*: dihydrolipoamide branched chain transacylase E2; *Pccb*: propionyl-CoA carboxylase subunit beta; *Aldh6a1*: aldehyde dehydrogenase family 6, subfamily A1; *Mcee*: methylmalonyl-CoA epimerase; *Mmmut*: methylmalonyl-CoA mutase; *Acads*: acyl-CoA dehydrogenase short chain; *Echs1*: enoyl-CoA hydratase, short chain 1; *Fh1*: fumarate hydratase 1; *Sdhd*: succinate dehydrogenase complex subunit D; *Sdhb*: succinate dehydrogenase complex iron sulfur subunit B; *Sdha*: succinate dehydrogenase complex flavoprotein subunit A; *Sdhc*: succinate dehydrogenase complex subunit C; *Mdh1*: malate dehydrogenase 1; *Acsm3*: acyl-CoA synthetase medium-chain family member 3; *HADH*: hydroxyacyl-CoA dehydrogenase; *Bdh1*: 3-hydroxybutyrate dehydrogenase 1; *Oxct1*: 3-oxoacid CoA-transferase 1; *Acat1*: acetyl-CoA acetyltransferase 1; *Cs*: citrate synthase; *Acly*: ATP citrate lyase; *Idh3g*: isocitrate dehydrogenase (NAD (+)) 3 non-catalytic subunit gamma; *Idh3a*: isocitrate dehydrogenase (NAD (+)) 3 catalytic subunit alpha; *Idh3b*: isocitrate dehydrogenase (NAD (+)) 3 non-catalytic subunit beta.

A total of 69 968 high-quality cells was identified with a mean of 11 661 cells per sample and the mean reads per cell was 26 665. These cells were partitioned into partitioned into 19 distinct clusters (C0–C18) (Fig. S10a). Based on canonical marker gene expression, these clusters were annotated to 13 unique cell types (Fig. 5a). Paneth cells (C0) accounted for the largest proportion of identified cells, followed by colonocytes (C3 and C4), secretory cells (C1 and C8), and colonic stem cells (C2 and C6) (Fig. S10b). We further investigated marker genes across different cell types. Specifically, for Myofibroblast, *Cdk4* and *Cdmn* were identified as marker genes; *Fer1l6* served as a marker for goblet cells, whereas the *Lgr5* was specifically enriched in colonic stem cells. Additionally, *Ccnd1* and *Ccnd2* showed remarkable enrichment in regenerative epithelial cells (Fig. 5b). Beyond these proliferation markers, *Ctnnb1* was also annotated as a marker gene for this population. *Ctnnb1* encodes the core transcriptional effector of the Wnt signaling pathway, which serves as a master upstream regulator of *Ccnd1* and *Ccnd2*. The co-markers of *Ctnnb1* with its downstream targets further supports the identity of this cluster as a regenerative epithelial population actively involved in Wnt-driven crypt regeneration and growth (Fig. 5b). Given that CON mice exhibited more mature colonic crypt morphology (deeper crypts) compared to the MAL group (Fig. 1b), these findings suggest that MAL treatment impairs colon development of mice. We therefore focused on regenerative epithelial cell population that exhibit high proliferative activity and serve as the key interface for microbial metabolite interactions. Differential gene analysis revealed that MAL treatment strongly suppressed the gene expression in regenerative epithelial cells, with 2852 genes significantly down-regulated and only 77 genes up-regulated (*P* < .05) (Fig. S11). Then, functional enrichment and pathway analysis further revealed MAL treatment primarily disrupted pathways related to metabolism (Fatty acid metabolism, Citrate cycle (TCA cycle), Propanoate metabolism and Butanoate metabolism), environmental information processing (Wnt signaling pathway as the dominant pathway directly regulating cell cycle progression and proliferative activity), and cellular processes (Cell cycle, focal adhesion and adherens junction) (*P* < .05) (Fig. 5c). Finally, guided by the reduced microbial SCFA production in MAL-treated mice, we next examined host colonic epithelial pathways involved in fatty acid metabolism and energy generation to establish a coherent link between microbial metabolites and host epithelial utilization (Fig. 5d). This suppression targeted key enzymes in butanoate metabolism (e.g. *Acsm3, Acads, Echs1*) and propanoate metabolism (e.g. *Pccb, Aldh6a1*), thereby blocking the microbial synthesis of SCFAs and their subsequent conversion into cellular energy. This upstream impairment further disrupted downstream oxidative phosphorylation (via reduced expression of *Sdha-d* and *Idh3a-c*, critical for electron transport and TCA cycle function) and TCA cycle activity (suppressed *Cs, Acly, Mdh1*, and *Fh1*), preventing efficient ATP production from SCFA-derived acetyl-CoA. These above functional insights delineate the specific molecular cascades disrupted within the metabolic pathways.

**Figure 6:**
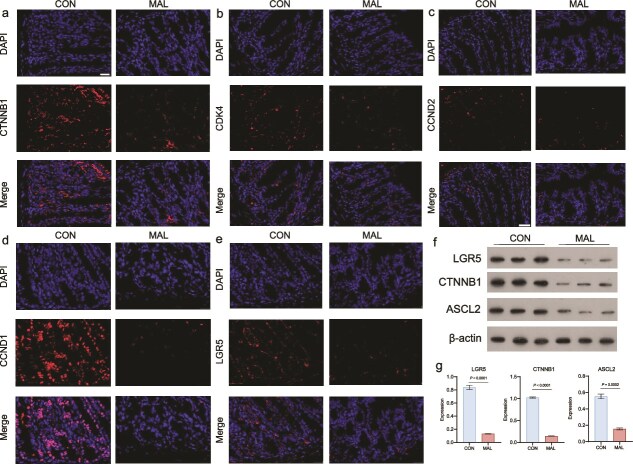
The expression levels of signature genes at the protein level between the CON and MAL groups. (a–e) immunofluorescence of CTNNB1, CDK4, CCND2, CCND1, and LGR5. The white scale bar indicates a length of 20 μm and corresponds to all images. (f) The protein expressions of the LGR5, CTNNB1 and ASCL2 detected by Western blot. (g) Quantification of Western blot bands. Statistical significance between the two groups was determined at *P* < .05.

The activity of cell cycle and proliferation pathways was further evaluated by measuring related protein expression levels via immunofluorescence staining and western blot (Fig. 6). The protein expression level of CTNNB1 (β-catenin) (*P* < .05) (Fig. 6a, Fig. S10), the core nuclear effector of Wnt signaling, was significantly reduced in the MAL group compared to the CON group. A parallel decrease was observed in key downstream proliferative portions including CCND1, CCND2 and CDK4 (*P* < .05) (Fig. 6b–d, Fig. S10). The expression of ASCL2 and LGR5 as two proteins critical for maintaining the self-renewal and differentiation capacity of gut stem cells at the crypt base was also suppressed by the MAL diet (*P* < .05) (Fig. 6e and f, Fig. S12). We initially performed Procrustes analysis, and the result revealed a significant concordance between the rumen metabolomic profile and the microbiome. In addition, metabolomic samples were also distinctly separated according to treatment groups.

## Discussion

Child malnutrition remains a global public health crisis, severely impeding human development and the achievement of sustainable development goals. Although progress has been made in addressing food shortages, nutritional imbalance—characterized primarily by poor dietary quality, insufficient protein and fat intake, and excessive carbohydrates—has emerged as a key driver of chronic stunting and related disorders. Here, by developing an isocaloric, nutritionally imbalanced diet, we successfully established a mouse model of chronic malnutrition in young animals, recapitulating the core features of this condition [10, 11]. Our study mechanistically demonstrates how dietary quality, rather than energy deficit alone, disrupts host homeostasis. Through integrated multi-omics analyses, combined with histological and biochemical assessments, we systematically unveil how nutritional imbalance perturbs the structure, function and stability of the gut microbial community, thereby impairing colonic development, metabolic homeostasis and signal transduction, ultimately leading to growth impairment.

Our findings of phenotype underscore the profound systemic impact of dietary quality on host growth and development. Mice fed the MAL diet exhibited not only significantly reduced body weight, body length, and liver mass, but also severe oxidative stress, systemic inflammation, and immune suppression, which likely exacerbated tissue damage and growth impairment [27, 28]. The marked decrease in IGF-1—a key anabolic hormone closely linked to growth—likely constitutes a major contributor to the observed growth impairment. This finding aligns with reports of low circulating IGF-1 levels in stunted individuals, further supporting its pivotal role [29, 30]. In addition, we identified a structural defect in the colon characterized by reduced crypt depth, suggesting that malnutrition-induced damage may originate in the gut and subsequently disseminate throughout the organism [8]. According to the results related to gut microbiome, the central finding of this study might establish the gut microbiota as a critical intermediary linking nutritionally imbalanced diets to host malnutrition. The 16 s rRNA sequencing revealed that the MAL diet induced a marked reduction in the diversity, richness, maturity, and stability of the murine gut microbial ecosystem. Due to the severe deficiency of multidimensional nutrients (protein, fat, and fiber) in the MAL diet, the sole carbon source deprived metabolically versatile bacteria of diverse metabolic substrates necessary for proliferation, leading to the loss of their dominant ecological niches and an inability to persist long-term in the gut. The previous studies have proved that diets rich in fiber and proteins could enhance microbial diversity and increase the abundance of secondary metabolic bacteria in the gut [31, 32]. In contrast, the absence of such dietary components suppresses these microbial features [33]. These findings align closely with our study observations. Together, these results underscore the indispensable role of dietary fiber and protein in sustaining a healthy gut microbial homeostasis. Building on this, functional profiling of the gut microbiota via metagenomics and targeted metabolomics uncovered the underlying mechanisms by which nutritional imbalance impairs microbial metabolic function. We observed a significant suppression of the *Dubosiella* genus, including species such as *Dubosiella_sp* and *Dubosiella_muris*, in MAL-fed mice. As an efficient producer of SCFAs, *Dubosiella* is implicated in beneficial microbial functions [34, 35]. This suggested that the MAL diet impairs key metabolic pathways by reducing the abundance of critical functional bacteria. As expected, our analyses indicated that MAL intervention elicited a concerted remodeling of microbial metabolism, predominantly affecting carbohydrate utilization, SCFAs biosynthesis, and amino acid metabolism negatively. Conversely, we also observed a significant enrichment of methane biosynthesis pathways in the gut microbiota of MAL-fed mice, although increased methane synthesis is often associated with energy loss in large livestock animals [36, 37], its impact on host energy deposition in a mouse model warrants further careful evaluation. Thus, the observed metabolic shift of methane might also partially be accounted for the reduced weight of mice in this study. In addition, we observed a convergent homogenization of gut microbial metabolic functions in independent animal models of gut disorders [38, 39] and suboptimal health [13, 40], which implied that malnutrition might inflict chronic injury to the host, particularly within the gut niche.

Leveraging scRNA-seq, we interrogated the mouse colon tissue at cellular resolution. Our analysis identified regenerating epithelial cells as the key responders to nutritional imbalance. Within the population of this cell, which possesses high proliferative potential [41, 42], we observed a broad suppression of gene expression programs in the MAL group. Specifically, this suppression occurred at two levels: first, the inhibition of cellular energy metabolism, evidenced by significantly downregulated expression of key SCFA-utilizing enzyme genes (such as *Acsm3* and *Aldh6a1*) in colonic epithelial cells. This subsequently led to suppressed expression of core genes in the TCA cycle (*Cs, Acly, Mdh1*) and oxidative phosphorylation pathway (*Sdha-d, Idh3a-c*), ultimately resulting in the cell’s inability to efficiently convert microbial-derived fuels into ATP required for development [43, 44]. In contrast, the suppression also targeted the canonical Wnt/β-catenin signaling pathway, which governs gut stem cell self-renewal and crypt proliferation [45]. The key effector CTNNB1 [46], its downstream target genes (CCND1, CCND2, CDK4) [47], and stem cell markers (LGR5, ASCL2) [48, 49] were consistently downregulated at both the transcriptional and protein levels. We next sought to delineate how reduced SCFA availability, resulting from microbial dysbiosis, mechanistically contributes to the suppression of Wnt/β-catenin signaling and the consequent colonic epithelial defects observed in our MAL model. Once taken up by colonocytes, SCFAs produced by microbiome are activated to their corresponding acyl-CoA derivatives by enzymes (involved in butyrate activation), which was significantly downregulated in the MAL group (Fig. 5d). These acyl-CoAs enter the TCA cycle via multiple metabolic enzymes to generate ATP required for β-catenin stabilization and nuclear translocation, thereby promoting its expression. Recent studies have provided direct evidence linking SCFAs to Wnt pathway activation: supplementation with SCFAs produced by *Ruminococcaceae* has been shown to promote intestinal stem cell proliferation by activating the Wnt/β-catenin signaling pathway [50]. In addition, SCFAs themselves can also function as histone deacetylase inhibitors, thereby altering chromatin accessibility and influencing the expression of genes involved in Wnt/β-catenin signaling [51]. Specific TCA cycle intermediates derived from SCFA metabolism, such as α-ketoglutarate, can also modulate Wnt/β-catenin signaling through DNA methylation and other mechanisms, thereby promoting intestinal epithelial regeneration [52, 53]. Thus, the SCFA deficiency induced by nutritional imbalance suppress Wnt/β-catenin signaling through impaired energy supply and reduced availability of SCFAs and their secondary metabolites as signaling molecules, ultimately impairing epithelial regeneration. In contrast, one study demonstrated that a *Bacillus subtilis*-derived metabolite enhances intestinal barrier integrity via the GADD45A-Wnt/β-catenin pathway, independently supporting our observation that this signaling cascade is critical for gut homeostasis [54]. In addition, a recent study demonstrated that β-glucuronidase-expressing *Lactobacillus reuteri* depletes the regenerative epithelial stem/progenitor pool during chemotherapy-induced enterotoxicity [55], whereas another reported that microbiota-sourced purines synergize with butyrate to support colonic energy metabolism and wound healing [56]. These findings align with our observations that microbial metabolic activities are closely linked to intestinal homeostasis and regenerative capacity.

In this study, it was a limitation that we used broad-spectrum antibiotics to deplete the gut microbiota prior to dietary intervention. Although this approach was chosen to standardize the microbial baseline, antibiotic treatment itself may transiently affect host physiology [57]. Because both CON and MAL groups underwent identical antibiotic treatment, any non-specific effects were equally distributed. Nevertheless, we acknowledge that future studies using germ-free or gnotobiotic mouse models would more definitively establish causality without antibiotic confounders [58]. Another limitation is the exclusive use of a mouse model. Caution is warranted when extrapolating to human malnutrition due to differences in gut microbiota composition [59], developmental timelines, and dietary responses between species [60, 61]. Future translational studies including human cohorts, intestinal organoids [62] and fecal microbiome transplantation from malnourished children are essential to validate our findings [63]. Finally, only female mice were used in this study. Although this minimized hormonal confounding, sex is known to influence microbiota composition and host metabolism [64]. The absence of male mice precludes assessment of sex-specific effects, and future studies should include both sexes.

In summary, this study demonstrates that dietary nutritional imbalance, independent of caloric intake, triggers a cascade of ecological disruptions within the gut microbiome—reducing diversity, destabilizing community structure, and suppressing beneficial metabolic outputs. These microbial perturbations, in turn, impair host colonic energy metabolism and suppress Wnt/β-catenin-mediated epithelial renewal, ultimately leading to stunted growth. Our findings underscore the importance of considering the gut microbial ecosystem as a key factor in early-life host development, and offer a framework for understanding how nutritional stress shapes host health through host–microbe interactions.

## Supplementary Material

Supplemental_Material_wrag135

## Data Availability

The 16S rRNA gene sequencing and metagenomic sequencing data generated in this study have been deposited in the NCBI Sequence Read Archive (SRA) under BioProject accession number PRJNA1449717 (https://www.ncbi.nlm.nih.gov/bioproject/?term=%20PRJNA1449717). The single-cell RNA sequencing data are available under GEO accession number GSE328617 (https://www.ncbi.nlm.nih.gov/geo/query/acc.cgi?acc=gse328617).
