# Supplementary material for: Early life nutritional imbalance impairs colonic epithelial regeneration through gut microbiota dysbiosis and metabolic suppression
Source: ISME J. 2026 May 29;20(1):wrag135. doi: 10.1093/ismejo/wrag135 (PMC13313320; doi:10.1093/ismejo/wrag135)
Supplement: Supplemental_Material_wrag135 [file supplemental_material_wrag135.docx]

Table S1 Nutritional components and ingredient of different mouse diet

|  | AIN-93G | | CON 13% Cellulose Diet (93G) | | MAL 4% Protein 2% Fat Diet | |
| --- | --- | --- | --- | --- | --- | --- |
| **Nutritional composition** | **gm%** | **kcal%** | **gm%** | **kcal%** | **gm%** | **kcal%** |
| Protein | 20.30% | 20.30% | 20.30% | 22.07% | 4.67% | 4.95% |
| Carbohydrate | 63.95% | 63.95% | 55.95% | 60.81% | 85.16% | 90.28% |
| Fat | 7.00% | 15.75% | 7.00% | 17.12% | 2.00% | 4.77% |
| Total |  | 100.00% |  | 100.00% |  | 100.00% |
| kcal/gm | 4.00 |  | 3.68 |  | 3.77 |  |
|  |  |  |  |  |  |  |
| **Ingredient** | **gm** | **kcal** | **gm** | **kcal** | **gm** | **kcal** |
| Casein | 200 | 800 | 200 | 800 | 46 | 184 |
| L-Cystine | 3 | 12 | 3 | 12 | 0.7 | 2.8 |
| Corn Starch | 397.486 | 1589.944 | 249.486 | 997.944 | 533.94 | 2135.76 |
| Maltodextrin | 132 | 528 | 200 | 800 | 200 | 800 |
| Sucrose | 100 | 400 | 100 | 400 | 107.7 | 430.8 |
| Cellulose | 50 | 0 | 130 | 0 | 50 | 0 |
| Soybean Oil | 70 | 630 | 70 | 630 | 20 | 180 |
| Vitamin Mix V10037 | 10 | 40 | 10 | 40 | 10 | 40 |
| Mineral Mix S10022G | 35 | 0 | 35 | 0 | 0 | 0 |
| Mineral Mix, w/o Ca & P | 0 | 0 | 0 | 0 | 13.4 | 0 |
| Calcium Phosphate, dibasic | 0 | 0 | 0 | 0 | 12 | 0 |
| Calcium Carbonate | 0 | 0 | 0 | 0 | 3.75 | 0 |
| TBHQ | 0.014 | 0 | 0.014 | 0 | 0.01 | 0 |
| Choline Bitartrate | 2.5 | 0 | 2.5 | 0 | 2.5 | 0 |
| Total | 1000 | 4000 | 1000 | 3680 | 1000 | 3773 |


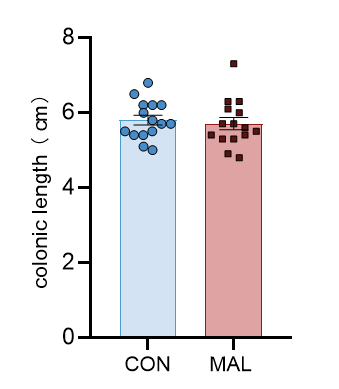


**Fig. S1** The difference of colonic length between the CON and MAL groups.

**
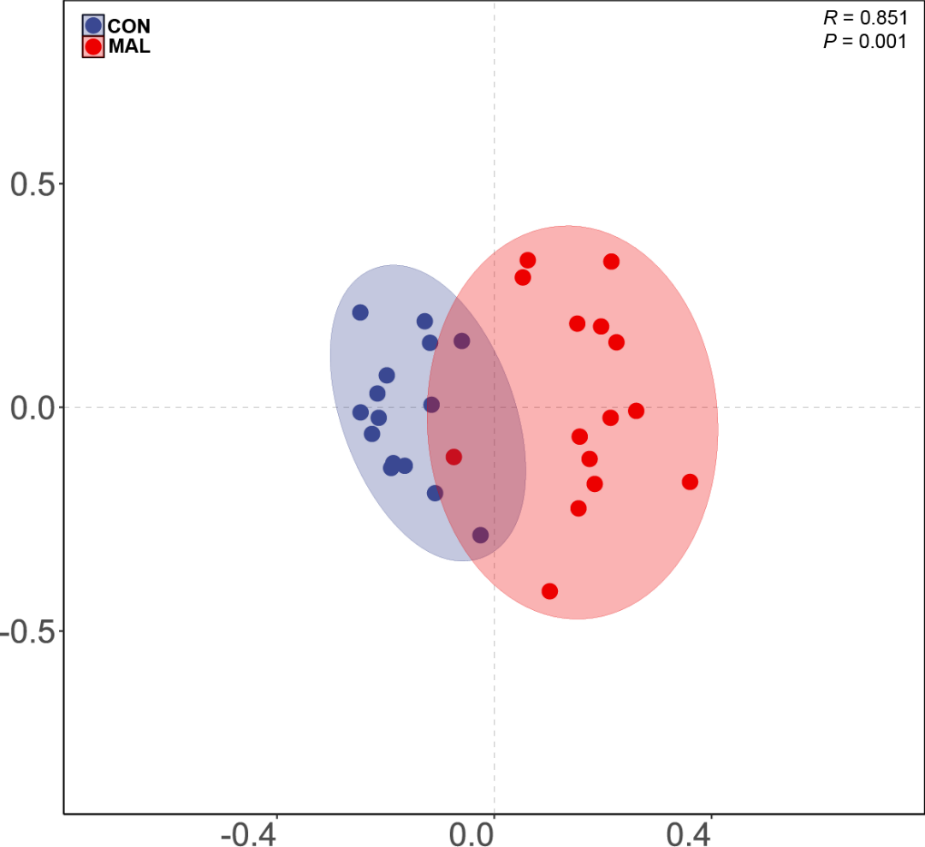
**

**Figure S2** The principal component analysis (PCA) of serum parameters in the CON and MAL groups.


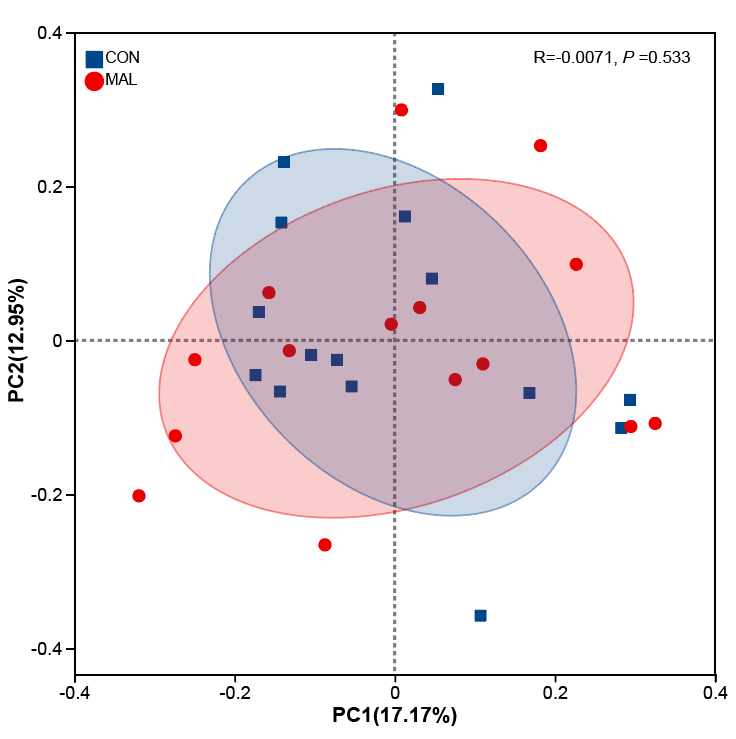


**Fig. S3** Principal coordinates analysis (PCoA) of gut microbial community structure in the CON and MAL groups at baseline prior to dietary intervention.


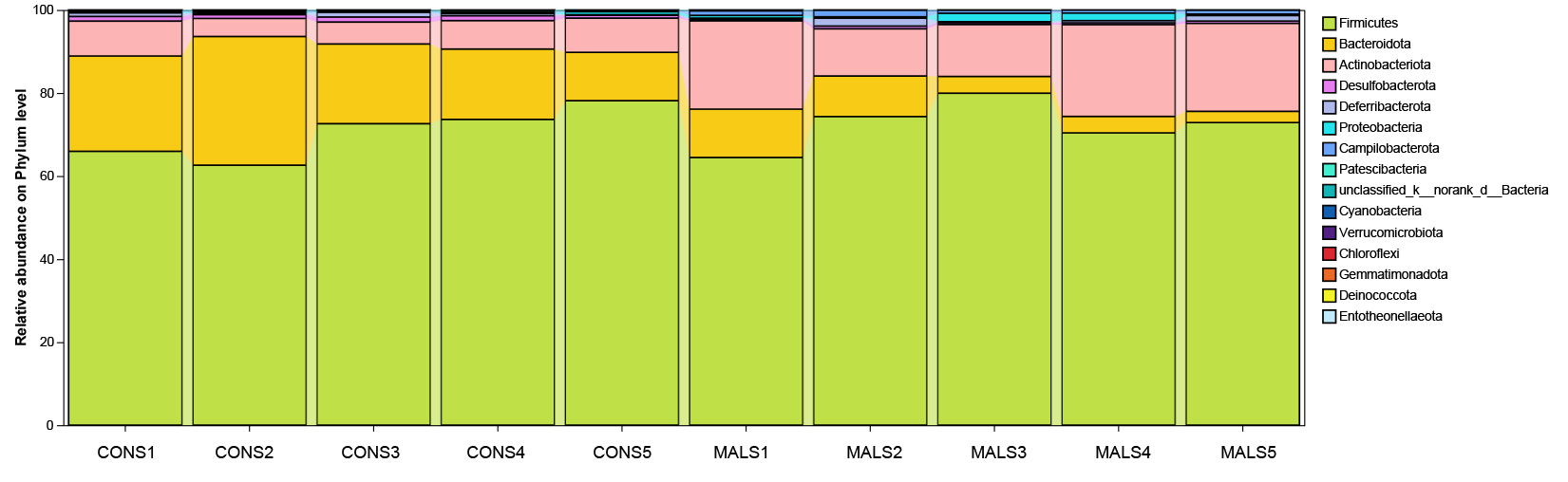


**Fig.** **S4** The composition of microbiota at the phylum level in the CON and MAL groups across the five timepoints.


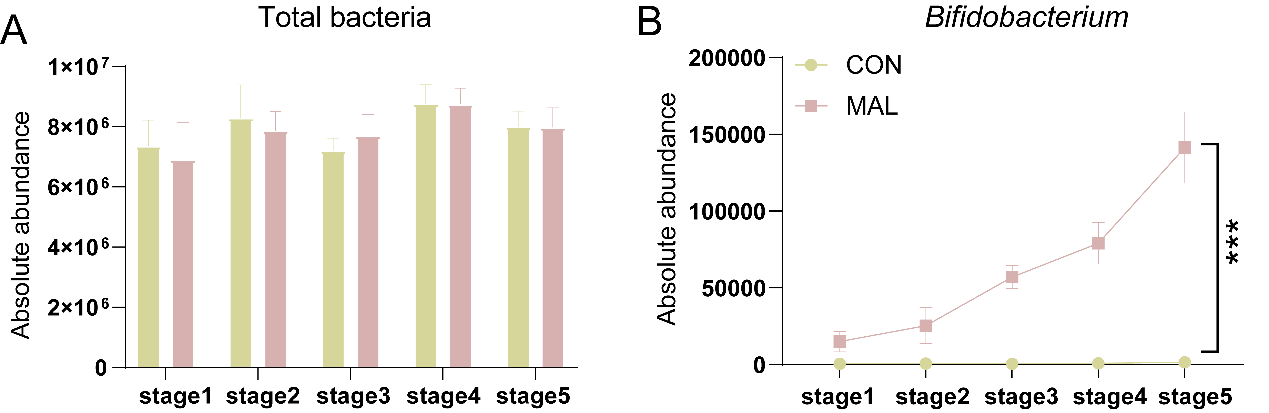


**Fig. S5** The absolute abundance of microbiota between the groups. **A** The absolute abundance of total bacteria. B The absolute abundance of *Bifidobacterium*.


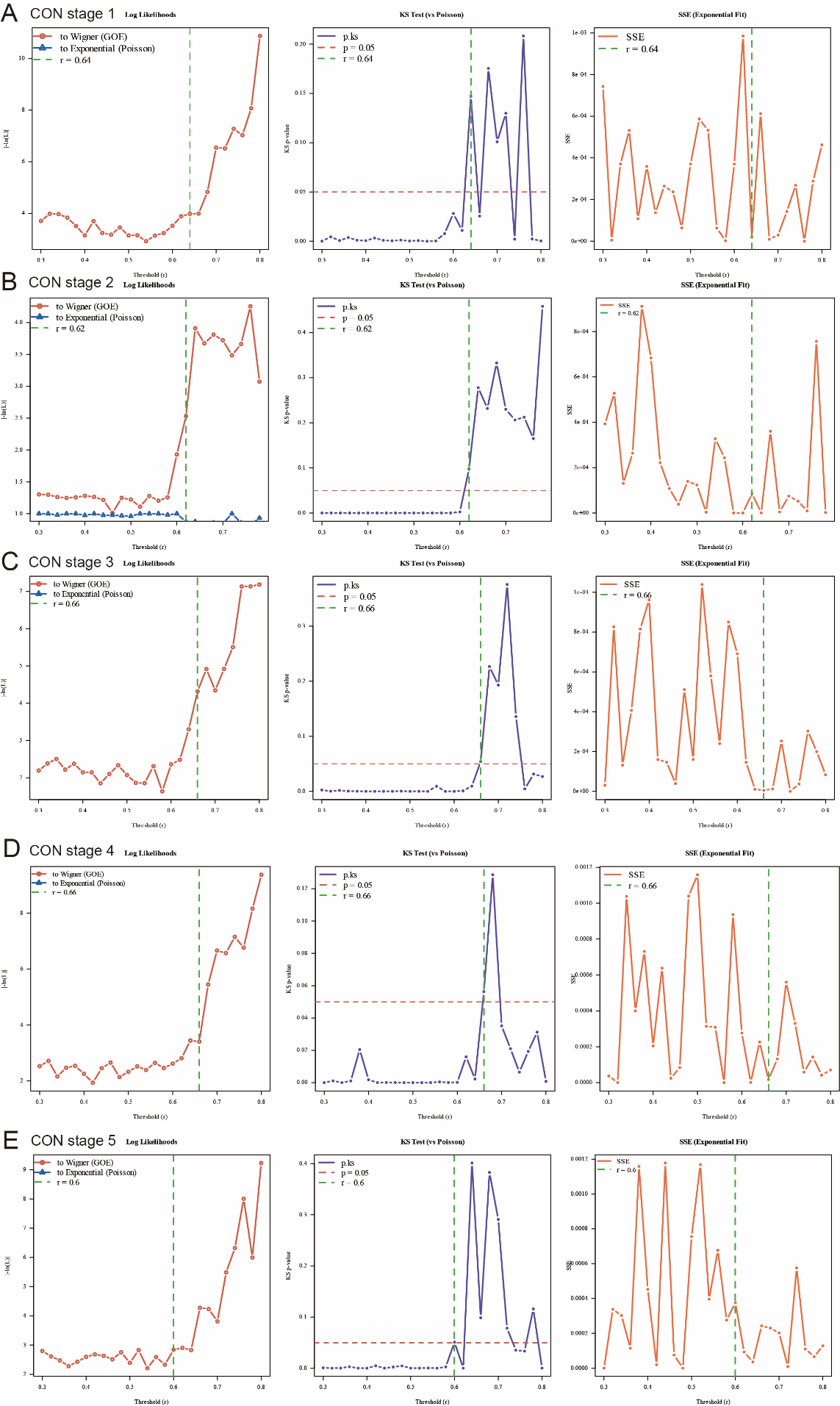


**Fig. S6** Random Matrix Theory (RMT) diagnostic plots for determining the correlation threshold across the timepoints of the CON group.


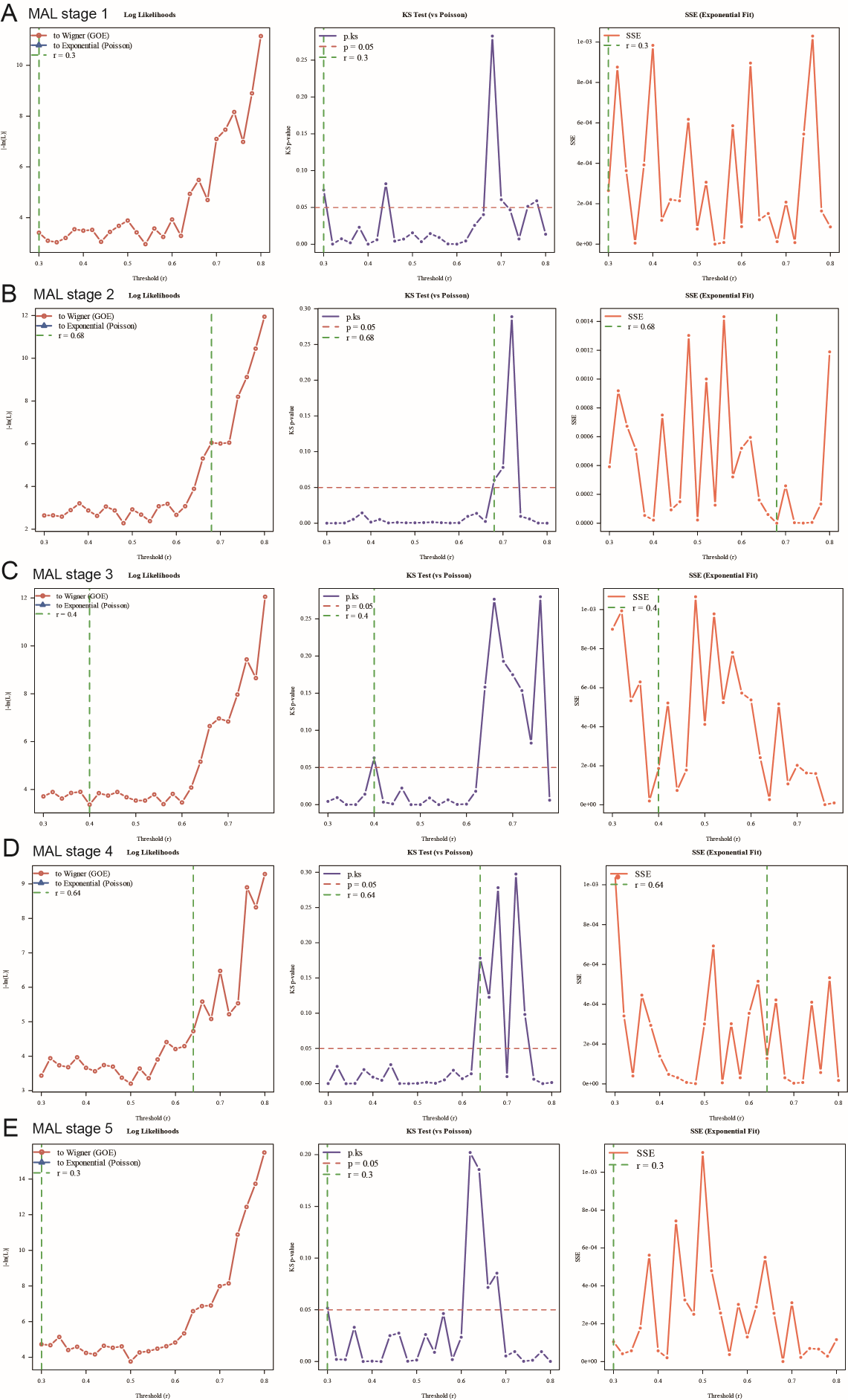


**Fig. S7** Random Matrix Theory (RMT) diagnostic plots for determining the correlation threshold across the timepoints of the MAL group.


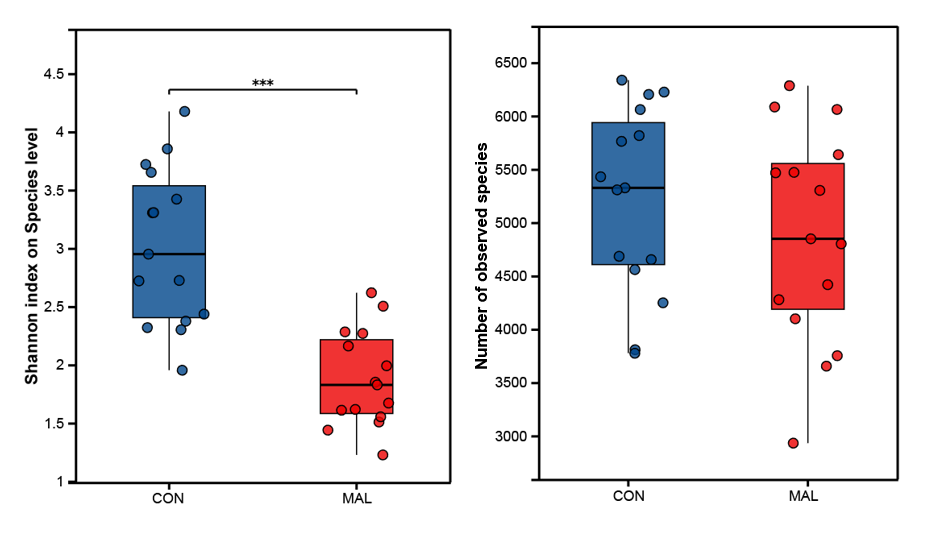


**Fig. S8** The comparisons of Shannon index and observed species between the CON and MAL groups.


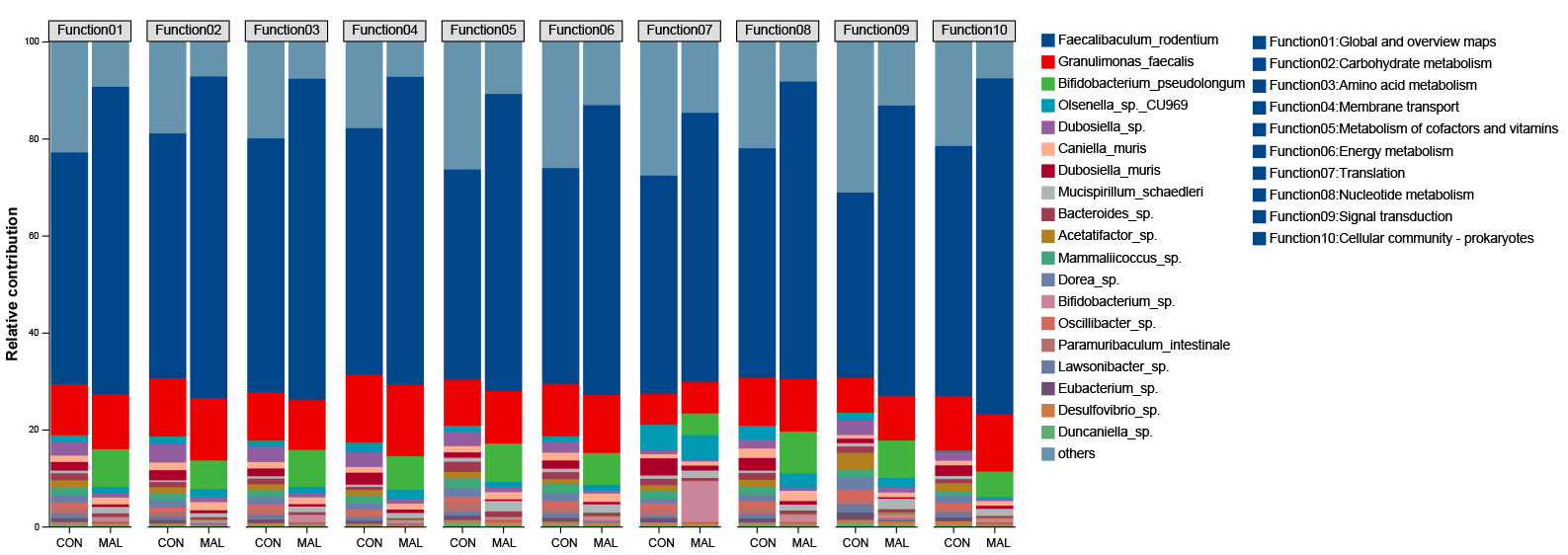


**Fig. S9** Species-level contributions to major microbial functions.


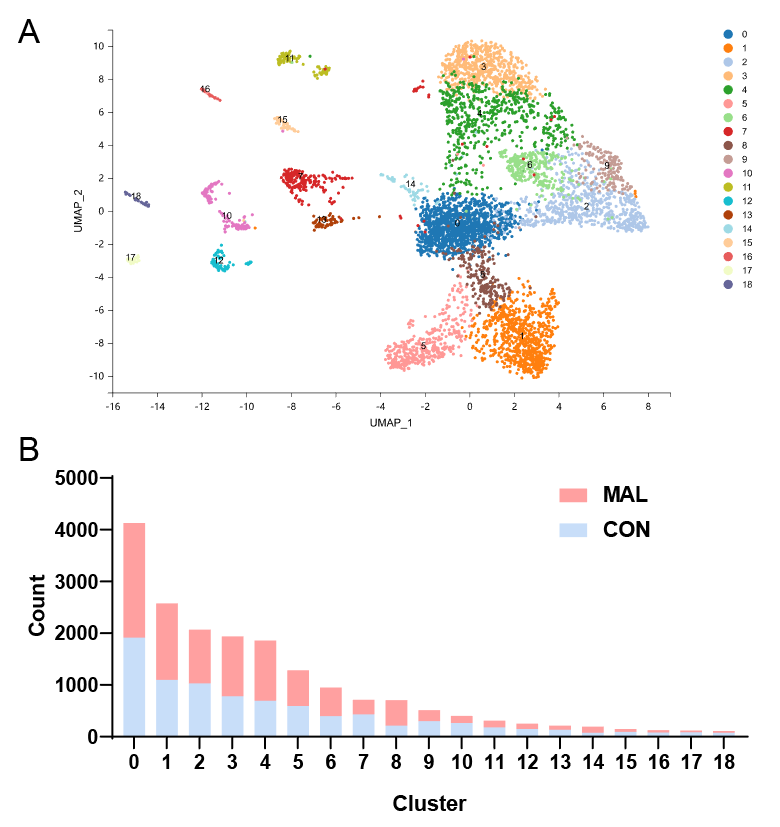


**Fig. S10** Basic information about cell clustering. **A** UMAP dimensionality reduction plot showing the distribution of cell clusters in the Con and MAL groups. **B** Comparative composition of cell types across the two groups**.**


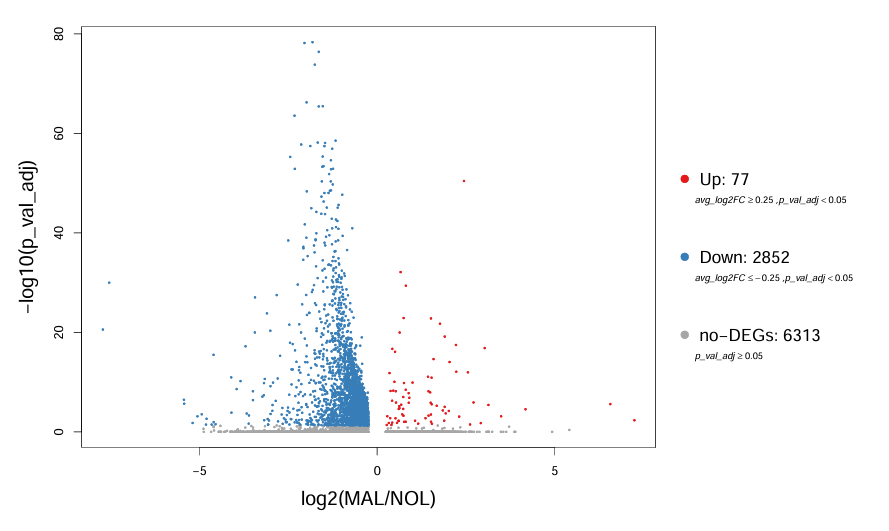


**Fig. S11** Volcano plot of differentially expressed genes (DEGs) in regenerative epithelial cells between the CON and MAL groups.


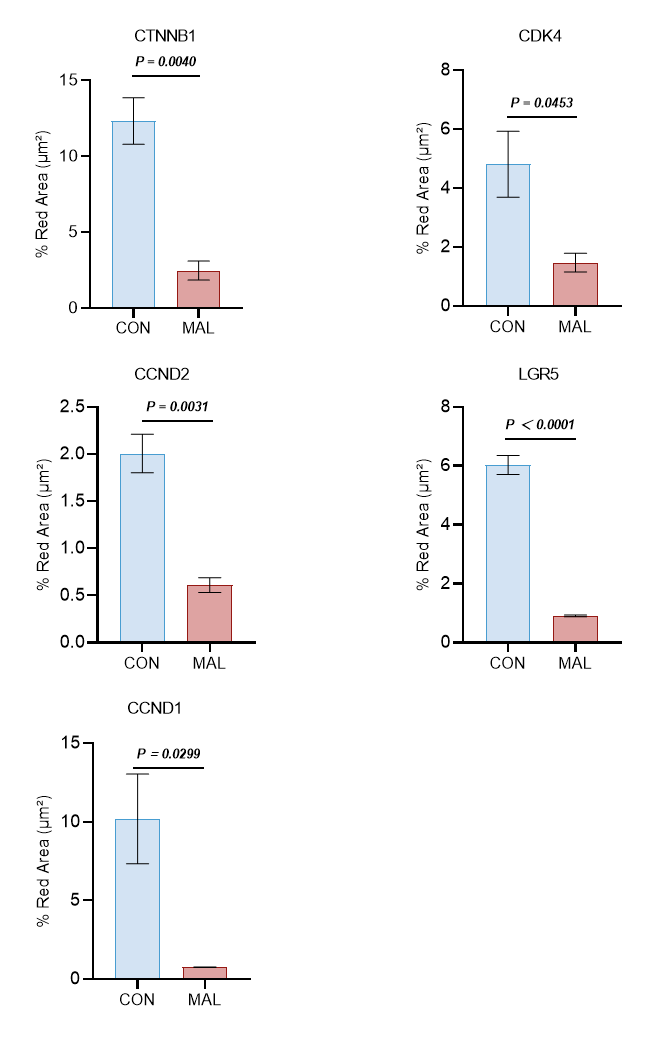


**Figure S12** The comparisons of red area in CTNNB1, CDK4, CCND2, LGR5 and CCND1.
